# A dataset of images and morphological profiles of 30 000 small-molecule treatments using the Cell Painting assay

**DOI:** 10.1093/gigascience/giw014

**Published:** 2017-01-07

**Authors:** Mark-Anthony Bray, Sigrun M Gustafsdottir, Mohammad H Rohban, Shantanu Singh, Vebjorn Ljosa, Katherine L Sokolnicki, Joshua A Bittker, Nicole E Bodycombe, Vlado Dančík, Thomas P Hasaka, Cindy S Hon, Melissa M Kemp, Kejie Li, Deepika Walpita, Mathias J Wawer, Todd R Golub, Stuart L Schreiber, Paul A Clemons, Alykhan F Shamji, Anne E Carpenter

**Affiliations:** Imaging Platform; Chemical Biology and Therapeutics Science Program; Center for the Development of Therapeutics; Cancer Program, Broad Institute of Harvard and MIT, Cambridge, MA, USA, 02142

**Keywords:** cellular morphology, high-content screening, image-based screening, phenotypic profiling, small-molecule library, U2OS

## Abstract

**Background:**

Large-scale image sets acquired by automated microscopy of perturbed samples enable a detailed comparison of cell states induced by each perturbation, such as a small molecule from a diverse library. Highly multiplexed measurements of cellular morphology can be extracted from each image and subsequently mined for a number of applications.

**Findings:**

This microscopy dataset includes 919 265 five-channel fields of view, representing 30 616 tested compounds, available at “The Cell Image Library” (CIL) repository. It also includes data files containing morphological features derived from each cell in each image, both at the single-cell level and population-averaged (i.e., per-well) level; the image analysis workflows that generated the morphological features are also provided. Quality-control metrics are provided as metadata, indicating fields of view that are out-of-focus or containing highly fluorescent material or debris. Lastly, chemical annotations are supplied for the compound treatments applied.

**Conclusions:**

Because computational algorithms and methods for handling single-cell morphological measurements are not yet routine, the dataset serves as a useful resource for the wider scientific community applying morphological (image-based) profiling. The dataset can be mined for many purposes, including small-molecule library enrichment and chemical mechanism-of-action studies, such as target identification. Integration with genetically perturbed datasets could enable identification of small-molecule mimetics of particular disease- or gene-related phenotypes that could be useful as probes or potential starting points for development of future therapeutics.

## Data Description

### Background

High-throughput quantitative analysis of cellular image data has led to critical insights across many fields in biology [1, 2]. While microscopy has enriched our understanding of biology for centuries, only recently has robotic sample preparation and microscopy equipment become widely available, together with large libraries of chemical and genetic perturbations. Concurrently, the advent of high-throughput imaging has also become an engine for pharmacological screening and basic research by allowing multiparametric image-based interrogation of physiological processes at a large scale [3, 4].

A typical imaging assay uses several fluorescent probes (or fluorescently tagged proteins) simultaneously with stain cells, each labeling distinct cellular components in each sample. In this way, the morphological characteristics (or “phenotype”) of cells, tissues, or even whole organisms can be examined, along with the concomitant changes induced by the perturbants of choice [5–7].

Phenotypic profiling has emerged as a powerful tool to discern subtle differences among treated samples in a relatively unbiased manner. In contrast to a screening strategy, where a usually limited number of features are quantified to select for a known cellular phenotype, profiling relies on collecting a large suite of per-cell morphological features and then using statistical analysis to uncover subtle morphological patterns (“signatures”) by which the perturbations can be characterized. The “Cell Painting” assay used for the dataset presented here uses fluorescent markers to broadly stain a number of cellular structures in high-throughput format, while automated software extracts the single-cell image-based morphological features. Further analysis then aggregates the data into multivariate profiles of these features to compare signatures among sample treatments.

The applications of image-based profiling are many and diverse. A dataset comprising small-molecule perturbations, as presented here, can be used for small-molecule library enrichment (to create smaller libraries while retaining high diversity of phenotypic impact) and small-molecule mechanism-of-action studies, including target identification. Integration of this dataset with datasets resulting from other types of perturbations (e.g., patient cell samples or genetically perturbed samples) enables identification of small-molecule mimetics of particular disease- or gene-related phenotypes that could be useful as probes or potential starting points for development of future potential therapeutics.

### Data acquisition protocol and quality control

**Figure 1:**
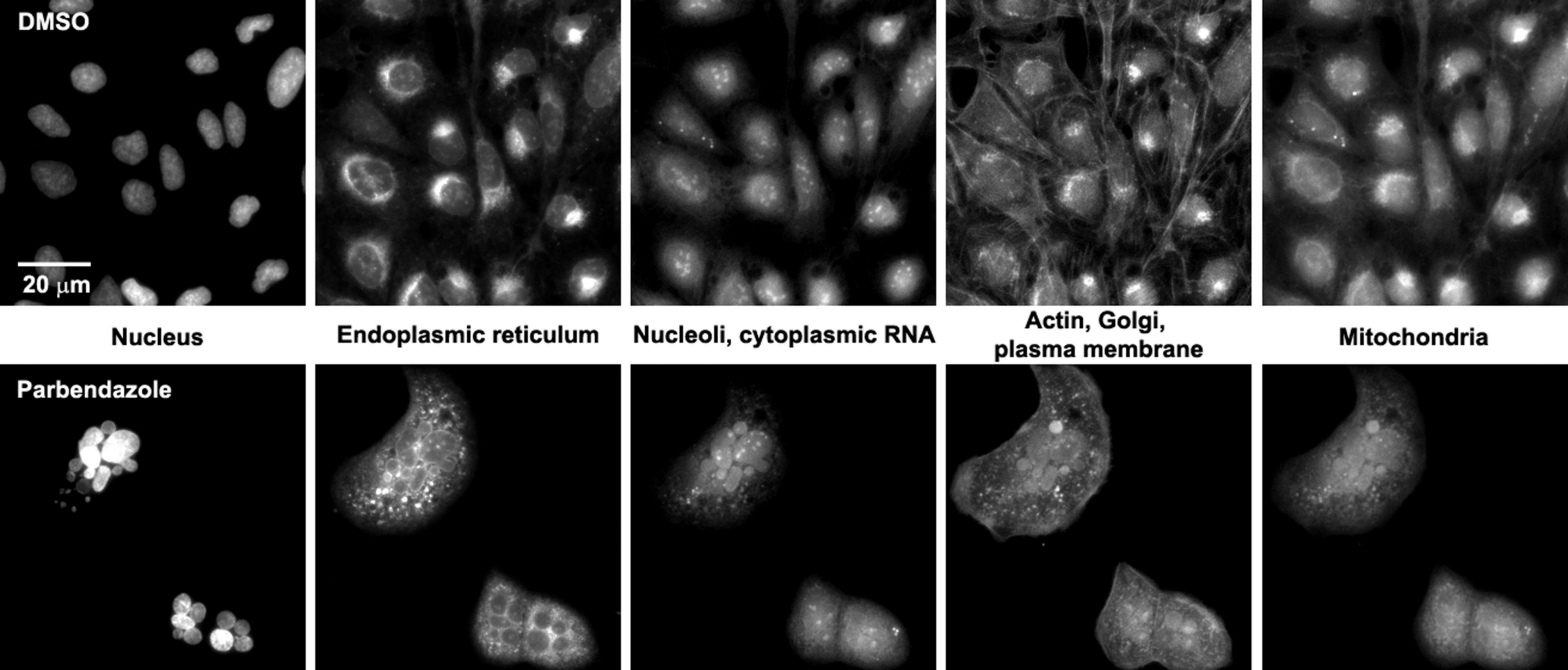
Sample images of U2OS cells from the small-molecule Cell Painting experiment. Images are shown from a DMSO well (negative control, top row) and a parbendazole well (bottom row). The columns display the 5 channels imaged in the Cell Painting assay protocol (see Table 1 for details about the stains and channels imaged).

To maximize the morphological information extracted from a single assay, we sought to “paint the cell” with as many distinct fluorescent morphological markers as possible simultaneously. Balancing technical and cost considerations, we developed the Cell Painting assay protocol, in which cells are stained for 8 major organelles and sub-compartments, using a mixture of 6 well-characterized fluorescent dyes suited for use in high throughput (Fig. 1) [8, 9].

The protocols for staining and imaging have been described in detail elsewhere [8, 9]. Briefly, U2OS cells were plated in 384-well plates, then treated with each of 30 616 compounds in quadruplicate. Of these compounds, 10 080 compounds came from the Molecular Libraries Small Molecule Repository (MLSMR) [10], 2260 were drugs, natural products, and small-molecule probes that are part of the Broad Institute known bioactive compound collection, 269 were confirmed screening hits from the Molecular Libraries Program (MLP), and 18 051 were novel compounds derived from diversity-oriented synthesis. Live cell staining was first performed to stain the mitochondria. After incubation, the cells were fixed with formaldehyde, permeabilized with Triton X-100, and stained with the remaining dyes to identify the nucleus (Hoechst), nucleoli and cytoplasmic RNA (SYTO 14), endoplasmic reticulum (concanavalin A), Golgi and plasma membrane (wheat germ agglutinin), and the actin cytoskeleton (phalloidin). Each of the 406 multi-well plates was imaged using an ImageXpress Micro XLS automated microscope (Molecular Devices, Sunnyvale, CA, USA), with 5 fluorescent channels at ×20 magnification, and 6 fields of view (sites) imaged per well (Table 1). Each image channel was then stored as a separate, grayscale image file in 16-bit TIF format. All raw image data are publicly available at “The Cell Image Library” (CIL) repository [11] and the Image Data Resource [12, 13].

**Table 1:** Details of dyes, stained cellular sub-compartments, and channels imaged in the Cell Painting assay

		Channel name	
Dye	Organelle or cellular component	CellProfiler	ImageXpress
Hoechst 33 342	Nucleus	DNA	w1
Concanavalin A/Alexa Fluor 488 conjugate	Endoplasmic reticulum	ER	w2
SYTO 14 green fluorescent nucleic acid stain	Nucleoli, cytoplasmic RNA	RNA	w3
Phalloidin/Alexa Fluor 594 conjugate, wheat germ agglutinin (WGA)/Alexa Fluor 594 conjugate	F-actin cytoskeleton, Golgi, plasma membrane	AGP	w4
MitoTracker Deep Red	Mitochondria	Mito	w5

The CellProfiler channel name refers to the name given by the software to each channel; this nomenclature also applies to the naming of the extracted morphological features. The ImageXpress channel name refers to the text in the raw image file name identifying the acquired wavelength. Please note that this protocol was later updated to use Phalloidin/Alexa Fluor 568 and WGA/Alexa Fluor 555, as described in [9].

The dataset available at *Giga*DB consists of the processed data derived from the acquired raw image data; the quantitative analysis of the images used a 3-step pipeline workflow created with the modular open-source software CellProfiler (Table 2; see also the Additional File and the “Availability of supporting data” section) [14]. First, an illumination pipeline estimated the heterogeneities in the spatial fluorescence distribution introduced by the microscope optics. This approximation was calculated on a per-plate basis for each channel and yielded a collection of illumination correction functions (ICFs) for later use in intensity correction; we have found that this approach not only aids in cell identification but also improves accuracy in signature classification [15]. Second, a quality control pipeline identified and labeled images with aberrations such as saturation artifacts and focal blur, as described previously (see also the Additional file) [16, 17]. Finally, a feature-extraction pipeline applied the ICFs to correct each channel, identified the nuclei, cell body, and cytoplasm, and extracted the morphological features for each cell, depositing the results into a database for downstream analysis (see the Additional file for a description of the extracted features). The extracted features include a broad array of cellular shape and adjacency statistics, as well as intensity and texture statistics that are measured in each channel. The pipelines, ICFs, and extracted morphological data are provided as a static snapshot in *Giga*DB [18] and in a *GigaScience* GitHub repository [19]. We note that the pipelines are configured for the archived CIL images; updates to the pipelines (and to the Cell Painting protocol in general) are provided online [20].

**Table 2:** Summary of the raw and intermediately processed data included in this Data Descriptor and nomenclature in the *Giga*DB and GitHub repositories

Data item	Location	Description
Raw fluorescence images	The Cell Image Library [11], GitHub: download_cil_images.sh	Five fluorescence channels, acquired at 6 fields of view per well at ×20 magnification (0.656 μm/pixel). The experiment comprises 406 plates in 384-well format (plates 24 277–26 796). We include a bash shell script to facilitate downloading the archives.
CellProfiler pipelines	GitHub: pipelines folder, GigaDB: pipelines.zip	CellProfiler software was used to correct for uneven illumination, perform quality control, and delineate cells into nuclei, cell body, and cytoplasmic sub-compartments and measure morphological features for each sub-compartment.
Illumination correction functions	GigaDB: <plate_ID>/illumination_correction_functions	An ICF is an estimation of the spatial illumination distribution introduced by the microscopy optics. There is 1 ICF per channel for each plate.
Quality control metadata	GigaDB: <plate_ID>/quality_control	Each field of view is assessed for the presence of 2 artifacts (focal blur and saturated objects), and assigned a label of 1 if present and 0 if not.
Extracted morphological features	GigaDB: <plate_ID>/extracted_features	A SQLite database comprising 4 tables (a) 1 per-image cellular statistic (e.g., cell count), (b) 3 per-cell cell tables, measuring size, shape, intensity, textural, and adjacency statistics for the nuclei, cytoplasm, and cell body.
Morphological profiles	GigaDB: <plate_ID>/profiles	Per-well averages of each extracted morphological feature computed across the cells.
Image curation statistics	GigaDB, GitHub: image_curation_statistics.csv	A summary of image statistics, such as the number of images, wells, and sites in the plates archived at The Cell Image Library, the number of sites with quality measures, and the number of wells with morphological profiles.
Chemical annotations	GigaDB, GitHub: chemical_annotations.csv	Chemical annotations including the compound names, SMILES, and PubChem identifiers (CID/SID)

<plate_ID> refers to the 5-digit plate ID assigned by the ImageXpress microscope system.

Many approaches exist to creating per-sample profiles based on the per-cell data from each replicate; we have found that producing profiles simply by averaging the cellular features across all cells for each well yielded good results in characterizing compounds [21]. These profiles are provided in *Giga*DB, along with a list of chemical annotations for the compounds applied. The downstream analysis of morphological profiling data is a field very much in flux at present; our own laboratory is developing an R package for this purpose [22] and has written a paper describing current data analysis strategies in the field [23].

### Potential uses

Phenotypic profiling provides a powerful means for assessing the biological impact of molecular or genetic perturbations, and for grouping sample treatments based on similarity. The applications are diverse and powerful; we only briefly summarize them here. The images and annotations provided in this Data Note have already been used in two published analyses from our own group: unsupervised clustering of a subset of 1601 bioactive compounds in a proof-of-principle study of compound mechanism of action [24, 25] and small-molecule library enrichment based on the full set of 30 616 small molecules, a study in which morphological profiles successfully selected compound subsets with higher-performance diversity than randomly selected compounds [8]. Other profiling applications include compound target identification, assessment of toxicity, and lead hopping. Further detail on applications of profiling, including those relevant to genetic perturbation datasets as opposed to the small molecule dataset described here, is available in a recent review [26].

This small-molecule dataset could also be used in more conventional applications; for example, if any of the morphological phenotypes in the experiment are of particular interest (e.g., mitochondrial structure or nucleolar size), the images and profiles can be re-mined, as in a conventional high-content screen, to produce “hit lists” of compounds that perturb those morphologies. The images and data can also be used as a look-up-table to identify morphological phenotypes produced by compounds that are deemed of interest in any particular high-throughput screen.

## Availability and requirements

- Project name: Supporting pipelines, scripts, and metadata for a Cell Painting dataset of 30 000 compounds.
- Project home page: https://github.com/gigascience/paper-bray2017
- Operating systems: Linux (for scripts), platform-independent (for pipelines)
- Programming language: Bash (for scripts)
- Other requirements: Unix (for scripts), CellProfiler 2.2.0 or later (for pipelines)
- License: GNU GPL v3
- Any restrictions to use by non-academics: none

## Availability of supporting data

The raw image data described in this article are available at “The Cell Image Library” repository as Plates 24 277–26 795 (http://www.cellimagelibrary.org/pages/project_20269, CIL: 24 277- CIL: 26 795) [11] as well as the Image Data Resource [13]. The remainder of the dataset supporting the results of this article is available in the *GigaScience* database, *Giga*DB (as a static snapshot), and GitHub repository [18, 19]. On *Giga*DB, all data relating to a plate are contained in sub-folders under a parent folder named with a unique 5-digit identifier for each plate. This includes illumination correction functions, metadata related to sample treatment and image quality control, extracted morphological features, and profiles (Table 2). Each of the plate folders has been packed as tape archives (TAR, .tar) before being compressed using GNU Gzip (.gz) and can be downloaded individually. Regrettably, not all the raw images could be retrieved from our archives, so not all plates have the full complement of 11 520 images; we have provided curation details listing the completeness of the archived data for each plate (Table 2). The GitHub repository also contains a bash shell script to facilitate downloading the entire CIL image set in batch, as well as image analysis pipelines and associated chemical annotation metadata. Updates to the pipelines (e.g., to accommodate updated software versions or updated versions of the protocol) can be found at our Cell Painting wiki [20]. An R package for the creation of well averages from single cell data can be found online [22, 27].

## Abbreviations

CIL: Cell Image Library; ICF: illumination correction functions; MLP: Molecular Libraries Program; MLSMR: Molecular Libraries Small Molecule Repository; WGA: wheat germ agglutinin.

## Competing interests

The authors declare that they have no competing interests.

## Funding

Research reported in this publication was supported in part by National Science Foundation CAREER DBI 1148823 (AEC) and National Institutes of Health R35 GM122547 (AEC).

## Author contributions

M.A.B. and A.E.C. drafted the manuscript. M.J.W., S.M.G., C.S.Y., J.A.B., T.R.G., A.E.C., A.F.S., S.L.S., and P.A.C. designed research. S.M.G., V.L., M.A.M., K.L.S., M.M.K., T.P.H., and J.A.B. performed research. M.J.W., K.L., V.L., N.E.B., M.A.B., V.D., A.E.C., A.F.S., S.L.S., P.A.C., S.S., M.H.R., and M.A.B. analyzed data. M.H.R. and S.S. reprocessed the dataset using updated pipelines and workflows. C.S.Y. served as a Project Manager.

## Supplementary Material

GIGA-D-16-00012_Original-Submission.pdf

GIGA-D-16-00012_Revision-1.pdf

GIGA-D-16-00012_Revision-2.pdf

Response-to-Reviewer-Comments_Original-Submission.pdf

Response-to-Reviewer-Comments_Revisions-1.pdf

Reviewer-1-Report-(Revision-1).pdf

Reviewer-1-Report-Original-Submission.pdf

Reviewer-1_Original-Submission-(attachement).pdf

Reviewer-2-Report-(Original-Submission).pdf
