# Supplementary material for: A dataset of images and morphological profiles of 30 000 small-molecule treatments using the Cell Painting assay
Source: Gigascience. 2017 Jan 7;6(12):1–5. doi: 10.1093/gigascience/giw014 (PMC5721342; doi:10.1093/gigascience/giw014)
Supplement: GIGA-D-16-00012_Revision-1.pdf [file giw014_giga-d-16-00012_revision-1.pdf]

Manuscript
Click here to download Manuscript gigascience-cell_painting.docx
Click here to view linked References

A dataset of images and morphological profiles of 30,000 small-molecule treatments using the Cell Painting assay

Mark-Anthony Bray[1] mbray@broadinstitute.org

Sigrun M. Gustafsdottir[2] (equal contributor) sigrun.gustafsdottir@gmail.com

Vebjorn Ljosa[1] (equal contributor) vebjorn@ljosa.com

Shantanu Singh[1] shsingh@broadinstitute.org

Katherine L. Sokolnicki[1] kate.sokolnicki@gmail.com

Joshua A. Bittker[2] jbittker@broadinstitute.org

Nicole E. Bodycombe[2] nemmith@gmail.com

Vlado Dančík[2] vdancik@broadinstitute.org

Thomas P. Hasaka[2] thasaka@gmail.com

C. Suk- Yee Hon[2] cindyhon@broadinstitute.org

Melissa M. Kemp[2] melissak.broad@gmail.com

Kejie Li[2] kejie.li@biogen.com

Deepika Walpita[2] walpitad@janelia.hhmi.org

Mathias J. Wawer[2] mwawer@broadinstitute.org

Todd R. Golub[3] golub@broadinstitute.org

Stuart L. Schreiber[2] schreiber@broadinstitute.org

Paul A. Clemons[2] pclemons@broadinstitute.org

Alykhan F. Shamji[2] ashamji@broadinstitute.org

Anne E. Carpenter[1]* anne@broadinstitute.org, http://www.broadinstitute.org/~anne/

[1] Imaging Platform, Broad Institute of Harvard and MIT, Cambridge, MA, USA

[2] Center for the Science of Therapeutics, Broad Institute of Harvard and MIT, Cambridge, MA, USA

[3] Cancer Program, Broad Institute of Harvard and MIT, Cambridge, MA USA

*To whom correspondence should be addressed

ABSTRACT

Background: Large-scale image sets acquired by automated microscopy of perturbed samples enable a detailed comparison of cell states induced by each perturbation, such as a small molecule from a diverse library. Highly multiplexed measurements of cellular morphology can be extracted from each image and subsequently mined for a number of applications.
Findings: This microscopy data set includes 919,874 five-channel fields of view representing 30,616 tested compounds, available at 'The Cell Image Library' repository. It also includes data files containing morphological features derived from each cell in each image, both at the single-cell level and population-averaged (i.e., per-well) level; the image analysis workflows that generated the morphological features are also provided. Quality-control metrics are provided as metadata, indicating fields of view that are out-of-focus or containing highly fluorescent material or debris. Lastly, chemical annotations are supplied for the compound treatments applied.
Conclusions: Because computational algorithms and methods for handling single-cell morphological measurements are not yet routine, the dataset serves as a useful resource for the wider scientific community applying morphological (image-based) profiling. The data set can be mined for many purposes, including small-molecule library enrichment and chemical mechanism-of-action studies, such as target identification. Integration with genetically-perturbed datasets could enable identification of small-molecule mimetics of particular disease- or gene-related phenotypes that could be useful as probes or potential starting points for development of future therapeutics.

KEYWORDS

phenotypic profiling, high-content screening, image-based screening, cellular morphology, small-molecule library, U2OS

DATA DESCRIPTION

Background

High-throughput quantitative analysis of cellular image data has led to critical insights across many fields in biology[1,2]. While microscopy has enriched our understanding of biology for centuries, only recently has robotic sample preparation and microscopy equipment become widely available, together with large libraries of chemical and genetic perturbations. Concurrently, the advent of high-throughput imaging has also become an engine for pharmacological screening and basic research, by allowing multiparametric image-based interrogation of physiological processes at a large scale[3,4].

A typical imaging assay uses several fluorescent probes (or fluorescently-tagged proteins) simultaneously to stain cells, each labeling distinct cellular components in each sample. In this way, the morphological characteristics (or "phenotype") of cells, tissues, or even whole organisms can be examined, along with the concomitant changes induced by the perturbants of choice[5–7].

Phenotypic profiling has emerged as a powerful tool to discern subtle differences among treated samples in a relatively unbiased manner. In contrast to a screening strategy, where a usually limited number of features are quantified to select for a known cellular phenotype, profiling relies on collecting a large suite of per-cell morphological features and then using statistical analysis to uncover subtle morphological patterns

(“signatures”) by which the perturbations can be characterized. The “Cell Painting” assay used for the dataset presented here uses fluorescent markers to broadly stain a number of cellular structures in high-throughput format, while automated software extracts the single-cell image-based morphological features. Further analysis then aggregates the data into multivariate profiles of these features to compare signatures among sample treatments.

The applications of image-based profiling are many and diverse. A dataset comprising small-molecule perturbations, as presented here, can be used for small-molecule library enrichment (to create smaller libraries while retaining high diversity of phenotypic impact) and small-molecule mechanism-of-action studies, including target identification. Integration of this dataset with datasets resulting from other types of perturbations (e.g., patient cell samples or genetically-perturbed samples) enables identification of small-molecule mimetics of particular disease- or gene-related phenotypes that could be useful as probes or potential starting points for development of future potential therapeutics.

Data acquisition protocol and quality control

To maximize the morphological information extracted from a single assay, we sought to “paint the cell” with as many distinct fluorescent morphological markers as possible simultaneously. Balancing technical and cost considerations, we developed the Cell Painting assay protocol in which cells are stained for eight major organelles and sub-compartments, using a mixture of six well-characterized fluorescent dyes suited for use in high-throughput (Fig. 1)[8,9].

The protocols for staining and imaging have been described in detail elsewhere[8,9]. Briefly, U2OS cells were plated in 384-well plates, then treated with each of 30,616 compounds in quadruplicate. Of these compounds, 10,162 compounds came from the Molecular Libraries Small Molecule Repository (MLSMR), 2,222 were drugs, natural products, and small- molecule probes that are part of the Broad Institute known bioactive compound collection, 274 were confirmed screening hits from the Molecular Libraries Program (MLP), and 19,137 were novel compounds derived from diversity-oriented synthesis. Live cell staining was first performed to stain the mitochondria using MitoTracker. After incubation, the cells were fixed with formaldehyde, permeabilized with Triton X-100, and stained with the remaining dyes to identify the nucleus (Hoechst), nucleoli and cytoplasmic RNA (SYTO 14), endoplasmic reticulum (concanavalin A), Golgi and plasma membrane (wheat germ agglutinin), and the actin cytoskeleton (phalloidin). Each of the 413 multi-well plates was imaged using an ImageXpress Micro XLS automated microscope (Molecular Devices), with five fluorescent channels at 20× magnification, and 6 fields of view (sites) imaged per well (Table 1). Each image channel was then stored as a separate, grayscale image file in 16-bit TIF format. All raw image data is publicly available at ‘The Cell Image Library’ repository[10].

The dataset available at GigaDB consists of the processed data derived from the acquired raw image data (Table 2; see also the Additional File and the “Availability of supporting data” section). The quantitative analysis of the images used a three-step workflow using the modular open-source software CellProfiler[11]. First, an illumination pipeline estimated the heterogeneities in the spatial fluorescence distribution introduced by the microscope optics. This approximation was calculated on a per-plate basis for each channel and yielded a collection of illumination correction functions (ICFs) for later use in intensity correction; we have found that this approach not only aids in cell identification but also improves accuracy in signature classification[12]. Second, a quality control pipeline identified and labeled images with aberrations such as saturation artifacts and focal blur as described previously[13,14] (see also Additional File). Finally, a feature-extraction pipeline applied the ICFs to correct each channel, identified the nuclei, cell body and cytoplasm, and extracted the morphological features for each cell, depositing the results into a database for downstream analysis (see Additional File for a

description of the extracted features). The extracted features include a broad array of cellular shape and adjacency statistics, as well as intensity and texture statistics that are measured in each channel. The pipelines, ICFs, and extracted morphological data are provided in the GigaDB repository. We note that the pipelines provided at GigaDB are configured for the archived CIL images; updates to the pipelines (and to the Cell Painting protocol in general) are provided at https://github.com/carpenterlab/2016_bray_natprot.

Many approaches exist to creating per-sample profiles based on the per-cell data from each replicate; we have found that producing profiles simply by averaging the cellular features across all cells for each well yielded good results in characterizing compounds[15]. These profiles are provided in GigaDB along with a listing of chemical annotations for the compounds applied. The downstream analysis of morphological profiling data is a field very much in flux at present; our own laboratory is developing an R package for this purpose at https://github.com/carpenterlab/cytomining-hackathon-wiki.

Potential uses

Phenotypic profiling provides a powerful means for assessing the biological impact of molecular or genetic perturbations, and for grouping sample treatments based on similarity. The applications are diverse and powerful; we only briefly summarize here. The images and annotations provided in this Data Note have already been used in two published analyses from our own group; unsupervised clustering of a subset of 1,601 bioactive compounds in a proof-of-principle study of compound mechanism of action (https://www.broadinstitute.org/bbbc/BBBC022/)[16] and small-molecule library enrichment based on the full set of 30,616 small molecules, a study in which morphological profiles successfully selected compound subsets with higher performance diversity than randomly-selected compounds[8]. Other profiling applications include compound target identification, assessment of toxicity, and lead hopping. Further detail on applications of profiling, including those relevant to genetic perturbation data sets as opposed to the small molecule data set described here, is available in a recent review [17].

This small-molecule data set could also be used in more conventional applications; for example, if any of the morphological phenotypes in the experiment are of particular interest (e.g., mitochondrial structure or nucleolar size), the images and profiles can be re-mined, as in a conventional high-content screen, to produce "hit lists" of compounds that perturb those morphologies. The images and data can also be used as a look-up-table to identify morphological phenotypes produced by compounds that are deemed of interest in any particular high-throughput screen.

AVAILABILITY AND REQUIREMENTS (applicable if code included)

Not applicable

AVAILABILITY OF SUPPORTING DATA

The raw image data described in this article is available at 'The Cell Image Library' repository as Plates 24277-26796 (http://www.cellimagelibrary.org/pages/project_20269, CIL: 24277- CIL: 26796)[10]. The remainder of the dataset supporting the results of this article is available in the GigaScience GigaDB repository [18]. All data relating to a plate are contained in sub-folders under a parent folder named with a unique 5-digit identifier for each plate. This includes illumination correction functions, metadata related to sample treatment and image quality control, extracted morphological features, and profiles (Table 2). Each of the plate folders has been packed as tape archives (TAR, .tar) before being compressed using GNU Gzip (.gz), and can be downloaded

individually. Regrettably, not all the raw images could be retrieved from our archives so not all plates have the full complement of 11,520 images; we have provided curation details listing the completeness of the archived data for each plate (Table 2). We have also provided a bash shell script to facilitate downloading the entire CIL image set in batch. Updates to the pipelines (e.g., to accommodate updated software versions or updated versions of the protocol) can be found at our Cell Painting wiki (https://github.com/carpenterlab/2016_bray_natprot). An R package for the creation of well averages from single cell data can be found at https://github.com/CellProfiler/cytominr. Any publication arising from the use of the deposited data must acknowledge the source of the dataset.

ETHICS APPROVAL AND CONSENT TO PARTICIPATE

Not applicable.

CONSENT FOR PUBLICATION

Not applicable.

COMPETING INTERESTS

The authors declare that they have no competing interests.

FUNDING

Research reported in this publication was supported in part by NSF CAREER DBI 1148823 (AEC).

AUTHOR CONTRIBUTIONS

MAB and AEC drafted the manuscript. MJW, SMG, CSYH, JAB, TRG, AEC, AFS, SLS, and PAC designed research. SMG, VL, MAM, KLS, MMK, TPH, and JAB performed research. MJW, KL, VL, NEB, MAB, VD, AEC, AFS, SLS, PAC, SS and MAB analyzed data. CSYH served as a Project Manager.

ACKNOWLEDGMENTS

The authors thank David Orloff and Willy Wong from ‘The Cell Image Library’ for their efforts in assisting in the upload and annotation of the image portion of the dataset, and Chris Hunter from GigaDB for validating data integrity of the image-derived portion of the dataset. We also thank Mohammad Hossein Rohban for expanding and contributing the compound annotations.

REFERENCES

1. Conrad C, Gerlich DW. Automated microscopy for high-content RNAi screening. J. Cell Biol. 2010;188:453–61.

2. Thomas N. High-content screening: a decade of evolution. J. Biomol. Screen. 2010;15:1–9.

3. Bickle M. The beautiful cell: high-content screening in drug discovery. Anal. Bioanal. Chem. 2010;398:219–26.

4. Boutros M, Heigwer F, Laufer C. Microscopy-Based High-Content Screening. Cell. 2015;163:1314–25.

5. Levsky JM, Singer RH. Gene expression and the myth of the average cell. Trends Cell Biol. 2003;13:4–6.

6. Snijder B, Pelkmans L. Origins of regulated cell-to-cell variability. Nat. Rev. Mol. Cell Biol. 2011;12:119–25.

7. Altschuler SJ, Wu LF. Cellular heterogeneity: do differences make a difference? Cell. 2010;141:559–63.

8. Wawer MJ, Li K, Gustafsdottir SM, Ljosa V, Bodycombe NE, Marton MA, et al. Toward performance-diverse small-molecule libraries for cell-based phenotypic screening using multiplexed high-dimensional profiling. Proc. Natl. Acad. Sci. U. S. A. 2014;111:10911–6.

9. Bray M-A, Singh S, Han H, Davis CT, Borgeson B, Gustafsdottir SM, et al. Cell Painting, an image-based assay for morphological profiling. Nat. Protoc.

10. Gustafsdottir SM, Ljosa V, Sokolnicki KL, Bittker JA, Bodycombe NE, Bray M-A, et al. Human U2OS cells - compound cell-painting experiment [Internet]. The Cell Image Library. 2015. Available from: http://www.cellimagelibrary.org/pages/project_20269

11. Kamentsky L, Jones TR, Fraser A, Bray M-A, Logan DJ, Madden KL, et al. Improved structure, function and compatibility for CellProfiler: modular high-throughput image analysis software. Bioinformatics. 2011;27:1179–80.

12. Singh S, Bray M-A, Jones TR, Carpenter AE. Pipeline for illumination correction of images for high-throughput microscopy. J. Microsc. 2014;256:231–6.

13. Bray M-A, Fraser AN, Hasaka TP, Carpenter AE. Workflow and metrics for image quality control in large-scale high-content screens. J. Biomol. Screen. 2012;17:266–74.

14. Bray M-A, Carpenter AE. Quality control for high-throughput imaging experiments using machine learning in CellProfiler. In: Paul A. Johnston PA, Trask OJ Jr, editors. Methods in Molecular Biology Series: High Content Imaging, Analysis and Screening: Applications in Basic Science and Drug Discovery. Humana Press (in revision).

15. Ljosa V, Caie PD, Ter Horst R, Sokolnicki KL, Jenkins EL, Daya S, et al. Comparison of methods for image-based profiling of cellular morphological responses to small-molecule treatment. J. Biomol. Screen. 2013;18:1321–9.

16. Gustafsdottir SM, Ljosa V, Sokolnicki KL, Anthony Wilson J, Walpita D, Kemp MM, et al. Multiplex cytological profiling assay to measure diverse cellular states. PLoS One. 2013;8:e80999.

17. Caicedo JC, Singh S, Carpenter AE. Applications in image-based profiling of perturbations. Curr. Opin. Biotechnol. 2016;39:134–42.

18. Bray M-A, Gustafsdottir SM, Ljosa V, Singh S, Sokolnicki KL, Bittker JA, et al. Supporting data for “A

dataset of images and morphological profiles of 30,000 small-molecule treatments using the Cell Painting assay." Gigascience repository. 2016. http://dx.doi.org/10.5524/100200

FIGURE LEGENDS

Figure 1: Sample images of U2OS cells from the small-molecule Cell Painting experiment. Images are shown from a DMSO well (negative control, top row) and a parbendazole well (bottom row). The columns display the five channels imaged in the Cell Painting assay protocol; see Table 1 for details about the stains and channels imaged.

TABLES AND CAPTIONS

Table 1: Details of dyes, stained cellular sub-compartments and channels imaged in the Cell Painting assay.

Dye	Organelle or cellular component	Channel name	
		CellProfiler	ImageXpress
Hoechst 33342	Nucleus	DNA	w1
Concanavalin A/Alexa Fluor 488 conjugate	Endoplasmic reticulum	ER	w2
SYTO 14 green fluorescent nucleic acid stain	Nucleoli, cytoplasmic RNA	RNA	w3
Phalloidin/Alexa Fluor 568 conjugate, wheat germ agglutinin (WGA)/Alexa Fluor 555 conjugate	F-actin cytoskeleton, Golgi, plasma membrane	AGP	w4
MitoTracker Deep Red	Mitochondria	Mito	w5

The CellProfiler channel name refers to the name given by the software to each channel; this nomenclature also applies to the naming of the extracted morphological features. The ImageXpress channel name refers to the text in the raw image file name identifying the acquired wavelength.

Table 2: Summary of the raw and intermediately processed data included in this Data Descriptor, and nomenclature in GigaDB.

Data item	Location	Description
Raw fluorescence images	The Cell Image Library[10], GigaDB: download_cil_images.sh	Five fluorescence channels, acquired at 6 fields of view per well at 20✕ magnification (0.656 µm/pixel). The experiment comprises 413 plates in 384-well format (Plates 24278-26794). We include a bash shell script to facilitate downloading the archives.
CellProfiler pipelines	GigaDB: pipelines.zip	CellProfiler software was used to correct for uneven illumination, perform quality control and delineate cells into nuclei, cell body and cytoplasmic sub-compartments and measure morphological features for each sub-compartment.
Illumination correction functions (ICFs)	GigaDB: <plate_ID>/illumination_correction_functions	An ICF is an estimation of the spatial illumination distribution introduced by the microscopy optics. There is one ICF per channel, for each plate.
Quality control metadata	GigaDB: <plate_ID>/quality_control	Each field of view is assessed for the presence of two artifacts (focal blur and saturated objects), and assigned a label of 1 if present, and 0 if not.
Extracted morphological features	GigaDB: <plate_ID>/extracted_features	Three data tables consisting of (a) per-image cellular statistics (e.g. cell count), (b) per-cell size, shape, intensity, textural and adjacency statistics measured for the nuclei, cytoplasm, and cell body, and (c) experimental metadata (e.g., compound applied).<br>Includes a MySQL dump file for importing the data tables into a MySQL database.
Morphological profiles	GigaDB: <plate_ID>/profiles	Per-well averages of each extracted morphological feature computed across the cells.
Image curation statistics	GigaDB: image_curation_statistics.csv	A summary of image statistics, such as the number of images, wells, and sites in the plates archived at The Cell Image Library, the number of sites with quality measures and the number of wells with morphological profiles.
Chemical annotations	GigaDB: chemical_annotations.csv	Chemical annotations including the compound names, SMILES, and PubChem identifiers (CID/SID)

<plate_ID> refers to the 5-digit plate ID assigned by the ImageXpress microscope system.

Figure 1 [Click here to download Figure Figure1.tif]

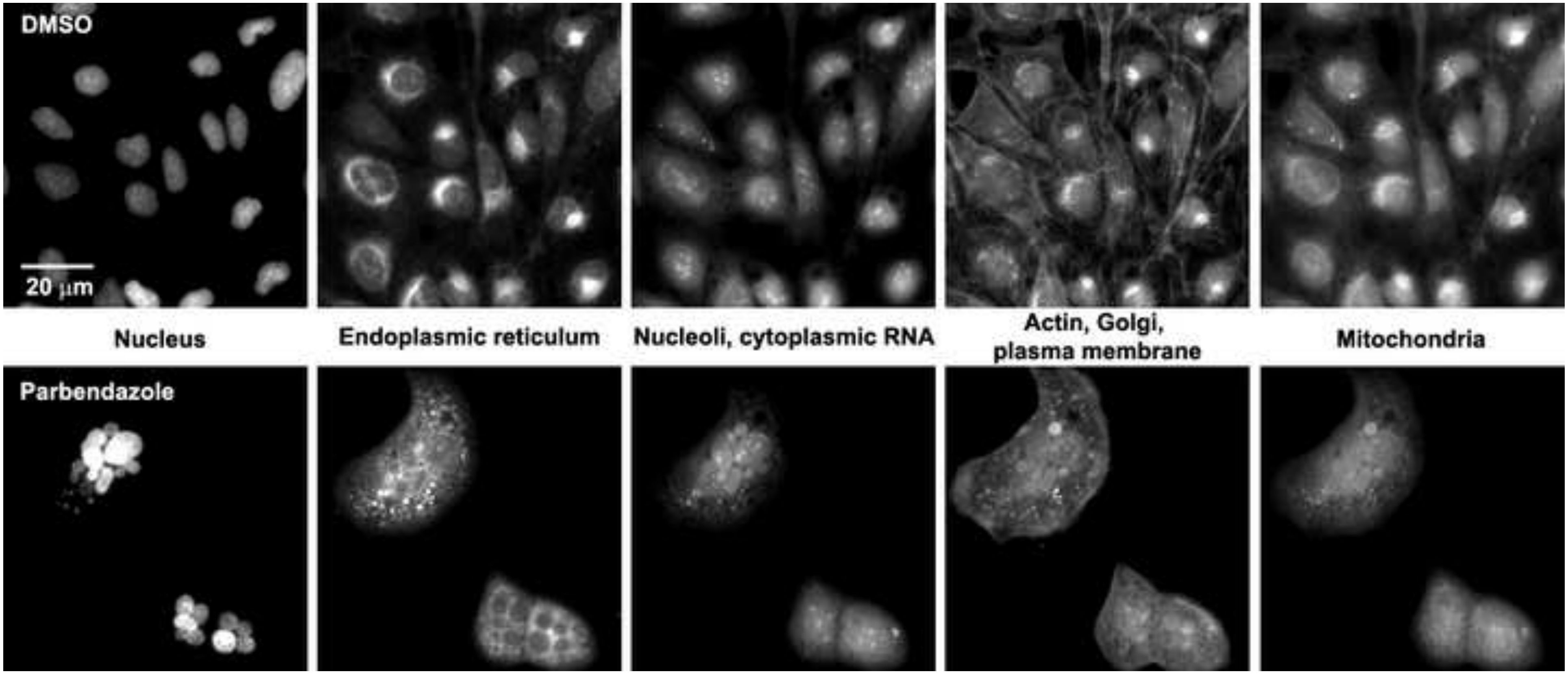

Workflows and processed data available at GigaDB repository

Click here to access/download
Supplementary Material
additional file.docx

BROAD INSTITUTE

415 Main Street
Cambridge, MA 02142
T 617-714-7000 F 617-714-8972
www.broadinstitute.org

Dear editors,

I am happy to submit our revised manuscript “A dataset of images and morphological profiles of 30,000 small-molecule treatments using the Cell Painting assay” (GIGA-D-16-00012). All substantial changes have been addressed in the manuscript and are described in the enclosed list of revisions.

The reviewers brought to our attention two items of note: that some of the image-derived data posted to GigDB had no corresponding Cell Painting images posted on ‘The Cell Image Library’ (CIL) repository; and that some of the GigaDB data may have become corrupted during uploading. We have addressed the former by contributing the omitted images to CIL and the latter by delivering file checksums to the GigaDB data managers. We are re-submitting the manuscript as we are awaiting the completion and verification of these two items.

I hope the manuscript can now be accepted for publication as a “Data Note” article in GigaScience. I look forward to hearing from you.

With kind regards,

Anne E. Carpenter, Ph.D.
Director, Imaging Platform
Broad Institute of Harvard and MIT
