# Supplementary material for: A dataset of images and morphological profiles of 30 000 small-molecule treatments using the Cell Painting assay
Source: Gigascience. 2017 Jan 7;6(12):1–5. doi: 10.1093/gigascience/giw014 (PMC5721342; doi:10.1093/gigascience/giw014)

Manuscript
Click here to download Manuscript GigaScience-CellPaintingRevision2.docx

A dataset of images and morphological profiles of 30,000 small-molecule treatments using the Cell Painting assay

Mark-Anthony Bray[1] mbray@broadinstitute.org

Sigrun M. Gustafsdottir[2] (equal contributor) sigrun.gustafsdottir@gmail.com

Vebjorn Ljosa[1] (equal contributor) vebjorn@ljosa.com

Shantanu Singh[1] shsingh@broadinstitute.org

Katherine L. Sokolnicki[1] kate.sokolnicki@gmail.com

Joshua A. Bittker[2] jbittker@broadinstitute.org

Nicole E. Bodycombe[2] nemmith@gmail.com

Vlado Dančík[2] vdancik@broadinstitute.org

Thomas P. Hasaka[2] thasaka@gmail.com

C. Suk-Yee Hon[2] cindyhon@broadinstitute.org

Melissa M. Kemp[2] melissak.broad@gmail.com

Kejie Li[2] kejie.li@biogen.com

Deepika Walpita[2] walpitad@janelia.hhmi.org

Mathias J. Wawer[2] mwawer@broadinstitute.org

Todd R. Golub[3] golub@broadinstitute.org

Stuart L. Schreiber[2] schreiber@broadinstitute.org

Paul A. Clemons[2] pclemons@broadinstitute.org

Alykhan F. Shamji[2] ashamji@broadinstitute.org

Anne E. Carpenter[1]* anne@broadinstitute.org, http://www.broadinstitute.org/~anne/

[1] Imaging Platform, Broad Institute of Harvard and MIT, Cambridge, MA, USA

[2] Center for the Science of Therapeutics, Broad Institute of Harvard and MIT, Cambridge, MA, USA

[3] Cancer Program, Broad Institute of Harvard and MIT, Cambridge, MA USA

*To whom correspondence should be addressed

ABSTRACT

Background: Large-scale image sets acquired by automated microscopy of perturbed samples enable a detailed comparison of cell states induced by each perturbation, such as a small molecule from a diverse library. Highly multiplexed measurements of cellular morphology can be extracted from each image and subsequently mined for a number of applications.

Findings: This microscopy data set includes 919,874 five-channel fields of view representing 30,616 tested compounds, available at ‘The Cell Image Library’ repository. It also includes data files containing morphological features derived from each cell in each image, both at the single-cell level and population-averaged (i.e., per-well) level; the image analysis workflows that generated the morphological features are also provided. Quality-control metrics are provided as metadata, indicating fields of view that are out-of-focus or containing highly fluorescent material or debris. Lastly, chemical annotations are supplied for the compound treatments applied.

Conclusions: Because computational algorithms and methods for handling single-cell morphological measurements are not yet routine, the dataset serves as a useful resource for the wider scientific community applying morphological (image-based) profiling. The data set can be mined for many purposes, including small-molecule library enrichment and chemical mechanism-of-action studies, such as target identification. Integration with genetically-perturbed datasets could enable identification of small-molecule mimetics of particular disease- or gene-related phenotypes that could be useful as probes or potential starting points for development of future therapeutics.

KEYWORDS

phenotypic profiling, high-content screening, image-based screening, cellular morphology, small-molecule library, U2OS

DATA DESCRIPTION

Background

High-throughput quantitative analysis of cellular image data has led to critical insights across many fields in biology[1,2]. While microscopy has enriched our understanding of biology for centuries, only recently has robotic sample preparation and microscopy equipment become widely available, together with large libraries of chemical and genetic perturbations. Concurrently, the advent of high-throughput imaging has also become an engine for pharmacological screening and basic research, by allowing multiparametric image-based interrogation of physiological processes at a large scale[3,4].

A typical imaging assay uses several fluorescent probes (or fluorescently-tagged proteins) simultaneously to stain cells, each labeling distinct cellular components in each sample. In this way, the morphological characteristics (or “phenotype”) of cells, tissues, or even whole organisms can be examined, along with the concomitant changes induced by the perturbants of choice[5–7].

Phenotypic profiling has emerged as a powerful tool to discern subtle differences among treated samples in a relatively unbiased manner. In contrast to a screening strategy, where a usually limited number of features are quantified to select for a known cellular phenotype, profiling relies on collecting a large suite of per-cell morphological features and then using statistical analysis to uncover subtle morphological patterns (“signatures”) by which the perturbations can be characterized. The “Cell Painting” assay used for the dataset presented here uses fluorescent markers to broadly stain a number of cellular structures in high-throughput format, while automated software extracts the single-cell image-based morphological features. Further analysis then aggregates the data into multivariate profiles of these features to compare signatures among sample treatments.

The applications of image-based profiling are many and diverse. A dataset comprising small-molecule perturbations, as presented here, can be used for small-molecule library enrichment (to create smaller libraries while retaining high diversity of phenotypic impact) and small-molecule mechanism-of-action studies, including target identification. Integration of this dataset with datasets resulting from other types of perturbations (e.g., patient cell samples or genetically-perturbed samples) enables identification of small-molecule mimetics of particular disease- or gene-related phenotypes that could be useful as probes or potential starting points for development of future potential therapeutics.

Data acquisition protocol and quality control

To maximize the morphological information extracted from a single assay, we sought to “paint the cell” with as many distinct fluorescent morphological markers as possible simultaneously. Balancing technical and cost considerations, we developed the Cell Painting assay protocol in which cells are stained for eight major organelles and sub-compartments, using a mixture of six well-characterized fluorescent dyes suited for use in high-throughput (Fig. 1)[8,9].

The protocols for staining and imaging have been described in detail elsewhere[8,9]. Briefly, U2OS cells were plated in 384-well plates, then treated with each of 30,616 compounds in quadruplicate. Of these compounds, 10,162 compounds came from the Molecular Libraries Small Molecule Repository (MLSMR)[10], 2,222 were drugs, natural products, and small- molecule probes that are part of the Broad Institute known bioactive compound collection, 274 were confirmed screening hits from the Molecular Libraries Program (MLP), and 19,137 were novel compounds derived from diversity-oriented synthesis. Live cell staining was first performed to stain the mitochondria. After incubation, the cells were fixed with formaldehyde, permeabilized with Triton X-100, and stained with the remaining dyes to identify the nucleus (Hoechst), nucleoli and cytoplasmic RNA (SYTO 14), endoplasmic reticulum (concanavalin A), Golgi and plasma membrane (wheat germ agglutinin), and the actin cytoskeleton (phalloidin). Each of the 413 multi-well plates was imaged using an ImageXpress Micro XLS automated microscope (Molecular Devices, Sunnyvale, CA, USA), with five fluorescent channels at 20× magnification, and 6 fields of view (sites) imaged per well (Table 1). Each image channel was then stored as a separate, grayscale image file in 16-bit TIF format. All raw image data is publicly available at ‘The Cell Image Library’ repository[11].

The dataset available at GigaDB consists of the processed data derived from the acquired raw image data; the quantitative analysis of the images used a three-step pipeline workflow created with the modular open-source software CellProfiler[12] (Table 2; see also the Additional File and the “Availability of supporting data” section). First, an illumination pipeline estimated the heterogeneities in the spatial fluorescence distribution introduced

by the microscope optics. This approximation was calculated on a per-plate basis for each channel and yielded a collection of illumination correction functions (ICFs) for later use in intensity correction; we have found that this approach not only aids in cell identification but also improves accuracy in signature classification[13]. Second, a quality control pipeline identified and labeled images with aberrations such as saturation artifacts and focal blur as described previously[14,15] (see also Additional File). Finally, a feature-extraction pipeline applied the ICFs to correct each channel, identified the nuclei, cell body and cytoplasm, and extracted the morphological features for each cell, depositing the results into a database for downstream analysis (see Additional File for a description of the extracted features). The extracted features include a broad array of cellular shape and adjacency statistics, as well as intensity and texture statistics that are measured in each channel. The pipelines, ICFs, and extracted morphological data are provided as a static snapshot in GigaDB[16] ands in a *Gigascience* GitHub repository[17]. We note that the pipelines are configured for the archived CIL images; updates to the pipelines (and to the Cell Painting protocol in general) are provided online[18].

Many approaches exist to creating per-sample profiles based on the per-cell data from each replicate; we have found that producing profiles simply by averaging the cellular features across all cells for each well yielded good results in characterizing compounds[19]. These profiles are provided in GigaDB along with a listing of chemical annotations for the compounds applied. The downstream analysis of morphological profiling data is a field very much in flux at present; our own laboratory is developing an R package for this purpose on our lab's GitHub page[20].

Potential uses

Phenotypic profiling provides a powerful means for assessing the biological impact of molecular or genetic perturbations, and for grouping sample treatments based on similarity. The applications are diverse and powerful; we only briefly summarize here. The images and annotations provided in this Data Note have already been used in two published analyses from our own group; unsupervised clustering of a subset of 1,601 bioactive compounds in a proof-of-principle study of compound mechanism of action (https://www.broadinstitute.org/bbbc/BBBC022/)[21] and small-molecule library enrichment based on the full set of 30,616 small molecules, a study in which morphological profiles successfully selected compound subsets with higher performance diversity than randomly-selected compounds[8]. Other profiling applications include compound target identification, assessment of toxicity, and lead hopping. Further detail on applications of profiling, including those relevant to genetic perturbation data sets as opposed to the small molecule data set described here, is available in a recent review [22].

This small-molecule data set could also be used in more conventional applications; for example, if any of the morphological phenotypes in the experiment are of particular interest (e.g., mitochondrial structure or nucleolar size), the images and profiles can be re-mined, as in a conventional high-content screen, to produce "hit lists" of compounds that perturb those morphologies. The images and data can also be used as a look-up-table to identify morphological phenotypes produced by compounds that are deemed of interest in any particular high-throughput screen.

AVAILABILITY AND REQUIREMENTS

- Project name: Supporting pipelines, scripts and metadata for cell painting data

- Project home page: https://github.com/gigascience/paper-bray2017
- Operating systems: Linux (for scripts), platform-independent (for pipelines)
- Programming language: Bash (for scripts)
- Other requirements: Unix (for scripts), CellProfiler 2.1.1 or later (for pipelines)
- License: GNU GPL v3

AVAILABILITY OF SUPPORTING DATA

The raw image data described in this article is available at 'The Cell Image Library' repository as Plates 24277-26796 (http://www.cellimagelibrary.org/pages/project_20269, CIL: 24277- CIL: 26796)[11]. The remainder of the dataset supporting the results of this article is available in the *GigaScience* GigaDB (as a static snapshot) and GitHub repositories [16,17]. On GigaDB, all data relating to a plate are contained in sub-folders under a parent folder named with a unique 5-digit identifier for each plate. This includes illumination correction functions, metadata related to sample treatment and image quality control, extracted morphological features, and profiles (Table 2). Each of the plate folders has been packed as tape archives (TAR, .tar) before being compressed using GNU Gzip (.gz), and can be downloaded individually. Regrettably, not all the raw images could be retrieved from our archives so not all plates have the full complement of 11,520 images; we have provided curation details listing the completeness of the archived data for each plate (Table 2). The GitHub repository also contains a bash shell script to facilitate downloading the entire CIL image set in batch, as well as image analysis pipelines and associated chemical annotation metadata. Updates to the pipelines (e.g., to accommodate updated software versions or updated versions of the protocol) can be found at our Cell Painting wiki[18]. An R package for the creation of well averages from single cell data can be found online[23].

COMPETING INTERESTS

The authors declare that they have no competing interests.

FUNDING

Research reported in this publication was supported in part by NSF CAREER DBI 1148823 (AEC).

AUTHOR CONTRIBUTIONS

MAB and AEC drafted the manuscript. MJW, SMG, CSYH, JAB, TRG, AEC, AFS, SLS, and PAC designed research. SMG, VL, MAM, KLS, MMK, TPH, and JAB performed research. MJW, KL, VL, NEB, MAB, VD, AEC, AFS, SLS, PAC, SS and MAB analyzed data. CSYH served as a Project Manager.

ACKNOWLEDGMENTS

The authors thank David Orloff and Willy Wong from 'The Cell Image Library' for their efforts in assisting in the upload and annotation of the image portion of the dataset, and Chris Hunter, Scott Edmunds and Peter Li from Gigascience for validating data integrity of the image-derived portion of the dataset and providing helpful

comments. We also thank Mohammad Hossein Rohban for expanding and contributing the compound annotations.

REFERENCES

1. Conrad C, Gerlich DW. Automated microscopy for high-content RNAi screening. J. Cell Biol. 2010;188:453–61.

2. Thomas N. High-content screening: a decade of evolution. J. Biomol. Screen. 2010;15:1–9.

3. Bickle M. The beautiful cell: high-content screening in drug discovery. Anal. Bioanal. Chem. 2010;398:219–26.

4. Boutros M, Heigwer F, Laufer C. Microscopy-Based High-Content Screening. Cell. 2015;163:1314–25.

5. Levsky JM, Singer RH. Gene expression and the myth of the average cell. Trends Cell Biol. 2003;13:4–6.

6. Snijder B, Pelkmans L. Origins of regulated cell-to-cell variability. Nat. Rev. Mol. Cell Biol. 2011;12:119–25.

7. Altschuler SJ, Wu LF. Cellular heterogeneity: do differences make a difference? Cell. 2010;141:559–63.

8. Wawer MJ, Li K, Gustafsdottir SM, Ljosa V, Bodycombe NE, Marton MA, et al. Toward performance-diverse small-molecule libraries for cell-based phenotypic screening using multiplexed high-dimensional profiling. Proc. Natl. Acad. Sci. U. S. A. 2014;111:10911–6.

9. Bray M-A, Singh S, Han H, Davis CT, Borgeson B, Gustafsdottir SM, et al. Cell Painting, a high-content image-based assay for morphological profiling using multiplexed fluorescent dyes. Nat. Protoc. 2016;11:1757–74.

10. Austin CP, Brady LS, Insel TR, Collins FS. NIH Molecular Libraries Initiative. Science. 2004;306:1138–9.

11. Gustafsdottir SM, Ljosa V, Sokolnicki KL, Bittker JA, Bodycombe NE, Bray M-A, et al. Human U2OS cells - compound cell-painting experiment [Internet]. The Cell Image Library. 2015. Available from: http://www.cellimagelibrary.org/pages/project_20269

12. Kamentsky L, Jones TR, Fraser A, Bray M-A, Logan DJ, Madden KL, et al. Improved structure, function and compatibility for CellProfiler: modular high-throughput image analysis software. Bioinformatics. 2011;27:1179–80.

13. Singh S, Bray M-A, Jones TR, Carpenter AE. Pipeline for illumination correction of images for high-throughput microscopy. J. Microsc. 2014;256:231–6.

14. Bray M-A, Fraser AN, Hasaka TP, Carpenter AE. Workflow and metrics for image quality control in large-scale high-content screens. J. Biomol. Screen. 2012;17:266–74.

15. Bray M-A, Carpenter AE. Quality control for high-throughput imaging experiments using machine learning in CellProfiler. In: Paul A. Johnston PA, Trask OJ Jr, editors. Methods in Molecular Biology Series: High Content Imaging, Analysis and Screening: Applications in Basic Science and Drug Discovery. Humana Press. *In press*

16. Bray, M, A; Gustafsdottir, S, M; Ljosa, V; Singh, S; Sokolnicki, K, L; Bittker, J, A; Bodycombe, N, E; Dančík, V; Hasaka, T, P; Hon, C, S; Kemp, M, M; Li, K; Walpita, D; Wawer, M, J; Golub, T, R; Schreiber, S, L; Clemons, P, A; Shamji, A, F; Carpenter, A, E (2016): Supporting data for "A dataset of images and

morphological profiles of 30,000 small-molecule treatments using the Cell Painting assay" GigaScience Database. http://dx.doi.org/10.5524/100200

17. Source code from “A dataset of images and morphological profiles of 30,000 small-molecule treatments using the Cell Painting assay.” [Internet]. GitHub. 2015 [cited 2016 Dec 15]. Available from: https://github.com/gigascience/paper-bray2017

18. Supporting data files, documentation, and updated tips for “Cell Painting, a high-content image-based assay for morphological profiling using multiplexed fluorescent dyes” [Internet]. GitHub. 2016 [cited 2016 Dec 6]. Available from: https://github.com/carpenterlab/2016_bray_natprot

19. Ljosa V, Caie PD, Ter Horst R, Sokolnicki KL, Jenkins EL, Daya S, et al. Comparison of methods for image-based profiling of cellular morphological responses to small-molecule treatment. J. Biomol. Screen. 2013;18:1321–9.

20. Cytomining Hackathon 2016 [Internet]. GitHub. 2016 [cited 2016 Dec 6]. Available from: https://github.com/carpenterlab/cytomining-hackathon-wiki

21. Gustafsdottir SM, Ljosa V, Sokolnicki KL, Anthony Wilson J, Walpita D, Kemp MM, et al. Multiplex cytological profiling assay to measure diverse cellular states. PLoS One. 2013;8:e80999.

22. Caicedo JC, Singh S, Carpenter AE. Applications in image-based profiling of perturbations. Curr. Opin. Biotechnol. 2016;39:134–42.

23. cytominer: library for mining patterns in perturbation data [Internet]. GitHub. 2015 [cited 2016 Dec 6]. Available from: https://github.com/CellProfiler/cytominer

FIGURE LEGENDS

Figure 1: Sample images of U2OS cells from the small-molecule Cell Painting experiment. Images are shown from a DMSO well (negative control, top row) and a parbendazole well (bottom row). The columns display the five channels imaged in the Cell Painting assay protocol; see Table 1 for details about the stains and channels imaged.

TABLES AND CAPTIONS

Table 1: Details of dyes, stained cellular sub-compartments and channels imaged in the Cell Painting assay.

Dye	Organelle or cellular component	Channel name	
		CellProfiler	ImageXpress
Hoechst 33342	Nucleus	DNA	w1
Concanavalin A/Alexa Fluor 488 conjugate	Endoplasmic reticulum	ER	w2
SYTO 14 green fluorescent nucleic acid stain	Nucleoli, cytoplasmic RNA	RNA	w3
Phalloidin/Alexa Fluor 568 conjugate, wheat germ agglutinin (WGA)/Alexa Fluor 555 conjugate	F-actin cytoskeleton, Golgi, plasma membrane	AGP	w4
MitoTracker Deep Red	Mitochondria	Mito	w5

The CellProfiler channel name refers to the name given by the software to each channel; this nomenclature also applies to the naming of the extracted morphological features. The ImageXpress channel name refers to the text in the raw image file name identifying the acquired wavelength.

Table 2: Summary of the raw and intermediately processed data included in this Data Descriptor, and nomenclature in the *Gigascience* GigaDB and GitHub repositories. <plate_ID> refers to the 5-digit plate ID assigned by the ImageXpress microscope system.

Data item	Location	Description
Raw fluorescence images	The Cell Image Library[11], GitHub: download_cil_images.sh	Five fluorescence channels, acquired at 6 fields of view per well at 20× magnification (0.656 μm/pixel). The experiment comprises 413 plates in 384-well format (Plates 24277-26796). We include a bash shell script to facilitate downloading the archives.
CellProfiler pipelines	GitHub: pipelines/ folder, GigaDB: pipelines.zip	CellProfiler software was used to correct for uneven illumination, perform quality control and delineate cells into nuclei, cell body and cytoplasmic sub-compartments and measure morphological features for each sub-compartment.
Illumination correction functions (ICFs)	GigaDB: <plate_ID>/illumination_correction_functions	An ICF is an estimation of the spatial illumination distribution introduced by the microscopy optics. There is one ICF per channel, for each plate.
Quality control metadata	GigaDB: <plate_ID>/quality_control	Each field of view is assessed for the presence of two artifacts (focal blur and saturated objects), and assigned a label of 1 if present, and 0 if not.
Extracted morphological features	GigaDB: <plate_ID>/extracted_features	Three data tables consisting of (a) per-image cellular statistics (e.g. cell count), (b) per-cell size, shape, intensity, textural and adjacency statistics measured for the nuclei, cytoplasm, and cell body, and (c) experimental metadata (e.g., compound applied).<br>Includes a MySQL dump file for importing the data tables into a MySQL database.
Morphological profiles	GigaDB: <plate_ID>/profiles	Per-well averages of each extracted morphological feature computed across the cells.
Image curation statistics	GigaDB, GitHub: image_curation_statistics.csv	A summary of image statistics, such as the number of images, wells, and sites in the plates archived at The Cell Image Library, the number of sites with quality measures and the number of wells with morphological profiles.
Chemical annotations	GigaDB, GitHub: chemical_annotations.csv	Chemical annotations including the compound names, SMILES, and PubChem identifiers (CID/SID)

Additional File: Workflows and processed data available at GigaDB and GitHub repositories

Availability of supporting data

The raw image data described in this article is available at 'The Cell: an Image Library' (CIL) repository as Plates 24277-26796 (http://www.cellimagelibrary.org/pages/project_20269, CIL: 24277- CIL: 26796)[1]. The remainder of the dataset supporting the results of this article is available in the *GigaScience* GigaDB (as a static snapshot)[2] and GitHub[3] repositories. On GigaDB, all data relating to a plate are contained in sub-folders under a parent folder named with a unique 5-digit identifier for each plate. This includes illumination correction functions, metadata related to sample treatment and image quality control, extracted morphological features, and profiles (Table 2 in the main manuscript). Each of the plate folders has been packed as tape archives (TAR, .tar) before being compressed using GNU Gzip (.gz), and can be downloaded individually. Regrettably, not all the raw images could be retrieved from our archives so not all plates have the full complement of 11,520 images; we have provided curation details listing the completeness of the archived data for each plate (Table 2 in the main manuscript). The GitHub repository also contains a bash shell script to facilitate downloading the entire CIL image set in batch, as well as image analysis pipelines and associated chemical annotation metadata. Updates to the pipelines (e.g., to accommodate updated software versions or updated versions of the protocol) can be found at our Cell Painting wiki (https://github.com/carpenterlab/2016_bray_natprot). An R package for the creation of well averages from single cell data can be found at https://github.com/CellProfiler/cytominr. Any publication arising from the use of the deposited data must acknowledge the source of the dataset.

Image data

The raw image data posted at CIL are archived by plate, with each plate's worth of data divided into five ZIP files, one for each Cell Painting channel. The ZIP file nomenclature is *<5-digit plate ID>-<channel ID>* where *<channel ID>* corresponds to:

- Hoechst : Hoechst 33342 channel (w1)
- ERSyto: Concanavalin A channel (w2)
- ERSytoBleed: SYTO 14 channel (w3)
- Ph_golgi: WGA/phalloidin channel (w4)
- Mito: MitoTracker Deep Red channel (w5)

CellProfiler pipelines

The raw image data are analyzed by the open-source software CellProfiler[4]. The GigaScience GitHub repository includes a set of three modular workflows (or "pipelines") for use with CellProfiler to handle three tasks: (a) illumination correction, (b) image quality control, and (c) morphological feature extraction. When a pipeline is loaded into CellProfiler, annotations can be viewed for each module of the pipeline, with details on the purpose of the module and considerations in making adjustments to the settings. The annotations may be found at the top of the settings, in the panel labeled "Module notes"; please refer to them for documentation relating to the image processing itself.

The illumination correction (illum.cppipe), quality control (quality_control.cppipe) and analysis (analysis.cppipe) pipelines were created with CellProfiler 2.1.1. All three pipelines are in the "pipelines" folder file in the

GigaScience GitHub repository and are adapted to work with the archived CIL images. Current and prior versions of CellProfiler may be found at http://cellprofiler.org/download.html.

The three pipelines provided in the GitHub repository make use of specialized Input modules which prompt for images from the user, collect metadata from the images, determine the correspondences between channels and assign user-specified names to the channels. We specifically chose to include these versions of the pipeline for compatibility with the CIL image set and to make them more convenient to adapt to a researcher's own images, which can be helpful for small-scale processing (~1,000 fields of view or less).

For large-scale processing, we recommend the use of the LoadData module rather than the Input modules; this module requires a comma-delimited file (CSV) as input. All the information produced by the Input modules is instead organized in this CSV. In order to convert the GigaScience pipelines for use at large-scale, follow the steps below for each pipeline:

- Open the pipeline in CellProfiler.
- Drag-and-drop the unzipped folder(s) of CIL images into the Images module. All five channels of images should be included in this operation. For the analysis pipeline, drag-and-drop the illumination correction functions as well (see the next section for more details on these files).
- Select *File* > *Export* > *Image Set Listing…* from the main menu, and enter the name of the CSV to be exported.
- To avoid ambiguity in image loading, we recommend opening the CSV in the spreadsheet editor of your choice (e.g. Excel) and removing all columns ***except*** the following:
    - Columns prefixed with "FileName" or "PathName"
    - Metadata_PlateID
    - Metadata_CPD_WELL_POSITION
    - Metdata_Site
- In CellProfiler, click the '+' button at the bottom-left, and from the dialog that appears, select the LoadData module located under the "File Processing" category. Click the "+ Add to Pipeline" button to insert it into the pipeline. Click 'Yes' to the legacy module prompt.
- If the LoadData module is not the first module in the pipeline, select the module and click the '^' or 'v' buttons at the bottom-left to move it into position.
- For the "Name of the file" setting, click the browse button and select the modified CSV created above.
- For the "Base image location" setting, select "None".
- (For the illumination correction pipeline only) For the "Group images by metadata?" setting, select "Yes". and select "PlateID" from the "Select metadata tags for grouping" listbox.

Note that we also maintain updated versions of the Cell Painting pipelines (not specifically adapted to the archived CIL images described in this paper, but kept up to date with latest versions of CellProfiler at our Cell Painting wiki at https://github.com/carpenterlab/2016_bray_natprot. In order to configure these updated pipelines for use with the CIL images, the Input modules need to be altered as follows:

- Metadata module
    - Change the metadata extraction method using the image filename as the source to `_(?P<CPD_WELL_POSITION>[a-p][0-9]{2})_s(?P<Site>[0-9])_w(?P<ChannelNumber>[0-9])`
    - Change the second metadata extraction method with folder as the source to `[\\/](?P<PlateID>[0-9]{5})`
- NamesAndTypes module:
    - For the rule criteria settings, set/confirm the proper channel-to-name correspondences, i.e, OrigDNA: Metadata ChannelNumber = 1, OrigER: Metadata ChannelNumber = 2, OrigRNA: Metadata ChannelNumber = 3, OrigAGP: Metadata ChannelNumber = 4, OrigMito: Metadata ChannelNumber = 5

    - For the metadata matching setting, select “PlateID” for the top row and “CPD_WELL_POSITION” for the second row, for all channels.
- (Illumination correction pipeline only) Groups module: For the metadata category setting, select ”PlateID”.

Our best practices for running CellProfiler pipelines at large-scale are described at https://github.com/CellProfiler/CellProfiler/wiki/Adapting-CellProfiler-to-a-LIMS-environment.

Illumination correction functions

Illumination heterogeneities introduced by the microscope optics can adversely affect intensity-based measurements and even impact cellular identification and segmentation. The illumination correction functions (ICF) included in this data set are post-hoc estimates of the microscope illumination distribution; heterogeneities are captured by the ICF and are used to correct the pixel intensities of the raw images. Each ICF is named according to the originating plate and associated channel: *<5-digit plate ID>_Illum<channel ID>.mat*, .e.g. *24278_IllumDNA.mat*; see Table 1 for the CellProfiler channel nomenclature.

The ICFs are in MATLAB (.mat) format to accommodate floating-point values, and are located in the “illumination_correction_functions” sub-folder in the archived file.

Extracted morphological features

The image-based morphological features extracted by CellProfiler are deposited into two data tables in comma-delimited text format (.csv); these are structured according to the following schema:

- An *image table* (“image.csv”) where each row corresponds to an image acquired at a unique field of view (site) and the columns contain the image data (e.g., the plate/well/site metadata, the name of the treatment condition, the filename of the original image, calculated segmentation thresholds etc). The metadata fields are as follows:
    - TableNumber: An integer index, used when multiple experiments are combined together into one table.
    - ImageNumber: An integer index; references each site (i.e., field of view) acquired.
    - Image_FileName_OrigER: Filename for the concanavalin A channel.
    - Image_FileName_OrigDNA : Filename for the Hoechst 33342 channel.
    - Image_FileName_OrigMito: Filename for the MitoTracker Deep Red channel.
    - Image_FileName_OrigAGP: Filename for the WGA/phalloidin channel.
    - Image_FileName_OrigRNA: Filename for the SYTO 14 channel.
    - Image_Metadata_PlateID: The 5-digit identifier given by the ImageXpress microscope labeling the plate.
    - Image_Metadata_CPD_WELL_POSITION: The well identifier as an alphanumeric label. Rows are labelled from ‘A’ to ‘P’, and columns are labelled from ‘01’ - ‘24’.
    - Image_Metadata_Site: The identifier for the fields of view in a well, numbered 1 to 6.
- An *object table* (“cells.csv”) in which each row represents an object (e.g., cells) from a given image and the columns contain the collected object measurements (e.g., area of the cell, intensity of DNA stain in the nucleus, location of the cell in the original image, etc). This table contains the TableNumber and ImageNumber indices described above, as well as an object index (column header: ObjectNumber) ntegers referencing each object (e.g., nucleus) identified in an image.
- A *metadata table* (“metadata.csv”) where each row corresponds to a particular plate/well combination and the columns contain the experimental metadata (e.g., the plate/well/site identifiers, the name of the treatment condition, etc). The metadata fields are as follows:

- Image_Metadata_PlateID: The 5-digit identifier given by the ImageXpress microscope labeling the plate.
- Image_Metadata_CPD_WELL_POSITION: The well identifier as an alphanumeric label. Rows are labelled from ‘A’ to ‘P’, and columns are labelled from ‘01’ - ‘24’.
- Image_Metadata_ASSAY_WELL_ROLE: Describes whether the well is a control (“mock”) or treatment (“compound”).
- Image_Metadata_BROAD_ID: The internal identifier from the Broad Institute’s compound management department.
- Image_Metadata_CPD_MMOL_CONC: The millimolar concentration from the compound stock plate; note that this is not the same as the final concentration used in the assay well.

A MySQL dump file is included to facilitate uploading the image and object tables to a MySQL database. More details on each feature can be found in the corresponding module documentation in CellProfiler.

The csv’s of morphological features are in the “extracted_features” folder sub-folder contained in the archived file.

Image quality control

To determine image data quality, we used a previously validated semi-automated workflow which labels images if they contain brightly fluorescing artifacts or are out-of-focus[5,6]. Briefly, a suite of whole-image measurements (e.g., intensity statistics, grayscale correlation and saturation percentage) are extracted from each image using CellProfiler. These image features form the basis for a supervised machine learning workflow using the open-source software package CellProfiler Analyst[7,8]. The training phase of the workflow consists of the researcher assembling a collection of example images, some of which are identified as containing artifacts and others as normal, i.e., artifact-free. Care is taken to ensure that the images originate across the entire experiment (as opposed to a narrow selection of wells or plates or well/column locations), to avoid overfitting. Based on this training set, a machine learning algorithm is applied to determine an initial classifier to discriminate aberrant from normal images. The software then presents the researcher with new samples of artifactual or artifact-free images, at which point they can correct errors and re-train the classifier. This workflow proceeds iteratively until the researcher is satisfied that the classifier achieves sufficient accuracy. The final classifier is then used to annotate all the images in the full experiment as being either saturated, blurred, or artifact-free.

The data tables of quality control annotations are in comma-delimited text format (.csv) in the “quality_control” folder sub-folder under the parent plate-ID folder contained in the archived file. In addition to the plate, well, and site metadata described above, the csv contains two metadata columns describing each field of view:

- Image_Metadata_isBlurry: Equal to 1 if the image is out-of-focus, 0 otherwise.
- Image_Metadata_isSaturated: Equal to 1 if image saturation artifacts are present, 0 otherwise.

In addition, the csv contains the values of the quality control metrics on which the classifier is based, for use if a researcher chooses to apply another classifier; these column headers are prefixed with “Image_ImageQuality_”.

Morphological profiles

We have previously published a comparison of methods for creating per-well profiles from the individual cell measurements from each image/site within the well[9]. Here, the profiles are given as a population average by computing the mean for each of the N morphological features across cells from all 6 sites per well, producing an N-dimensional data vector for each well.

The data tables of profiles are formatted as comma-delimited text (.csv) with the same feature nomenclature as the object table described above; they are located in the “profiles” sub-folder contained in the archived file.

Image curation statistics

A spreadsheet summarizing the curation statistics is included as comma-delimited text (.csv). The following items are listed for each plate:

- Plate ID: 5-digit identifier given by the ImageXpress microscope labeling the plate.
- Num_CIL_images: Total number of images for the plate hosted at The Cell Image Library (CIL).
- Num_CIL_wells: Total number of wells represented in the plate hosted at CIL which have >1 site (i.e., field of view) included.
- Num_CIL_complete wells: Total number of wells which have all sites included.
- Num_CIL_sites: Total number of sites which have >1 channel included.
- Num_CIL_complete_sites: Total number of sites which have all channels included.
- Num_QC_stats: Total number of sites for which quality control data is included.
- Num_blurry_sites: Total number of sites labelled as blurry/out-of-focus by the quality control workflow.
- Num_saturated_sites: Total number of sites labelled as containing saturation artifacts by the quality control workflow.
- Num_well_profiles: Total number of wells which have morphological profiles included in the GigaDB repository.

Chemical compound annotations

As mentioned above, each folder containing per-plate data also contains a corresponding table of treatment metadata. We have also included a csv containing metadata for many of the compounds from Broad Institute’s Chemical Biology Informatics Platform (CBIP), reference by BROAD_ID (the internal identifier from the Broad Institute’s compound management department) and including (where applicable) compound names, simplified molecular-input line-entry system annotations (SMILES), MLSMR sample identifiers, and PubChem compound identifiers (CID) and substance identifiers (SID). The latter two items are useful for querying the PubChem Compound Database (http://www.ncbi.nlm.nih.gov/pccompound).

References

1. Gustafsdottir SM, Ljosa V, Sokolnicki KL, Bittker JA, Bodycombe NE, Bray M-A, et al. Human U2OS cells - compound cell-painting experiment [Internet]. The Cell Image Library. 2015. Available from: http://www.cellimagelibrary.org/pages/project_20269

2. Bray M-A, Gustafsdottir SM, Ljosa V, Singh S, Sokolnicki KL, Bittker JA, et al. Supporting data for “A dataset of images and morphological profiles of 30,000 small-molecule treatments using the Cell Painting assay” [Internet]. GigaDB. 2016. Available from: http://gigadb.org/dataset/100200

3. Source code from “A dataset of images and morphological profiles of 30,000 small-molecule treatments using the Cell Painting assay.” [Internet]. GitHub. 2015 [cited 2016 Dec 15]. Available from: https://github.com/gigascience/paper-bray2017

4. Kamentsky L, Jones TR, Fraser A, Bray M-A, Logan DJ, Madden KL, et al. Improved structure, function and compatibility for CellProfiler: modular high-throughput image analysis software. Bioinformatics. 2011;27:1179–80.

5. Bray M-A, Fraser AN, Hasaka TP, Carpenter AE. Workflow and metrics for image quality control in large-scale high-content screens. J. Biomol. Screen. 2012;17:266–74.

6. Bray M-A, Carpenter AE. Quality control for high-throughput imaging experiments using machine learning in CellProfiler. In: Paul A. Johnston PA, Trask OJ Jr, editors. Methods in Molecular Biology Series: High Content Imaging, Analysis and Screening: Applications in Basic Science and Drug Discovery. Humana Press,;

7. Jones TR, Kang IH, Wheeler DB, Lindquist RA, Papallo A, Sabatini DM, et al. CellProfiler Analyst: data exploration and analysis software for complex image-based screens. BMC Bioinformatics. 2008;9:482.

8. Jones TR, Carpenter AE, Lamprecht MR, Moffat J, Silver SJ, Grenier JK, et al. Scoring diverse cellular morphologies in image-based screens with iterative feedback and machine learning. Proc. Natl. Acad. Sci. U. S. A. 2009;106:1826–31.

9. Ljosa V, Caie PD, Ter Horst R, Sokolnicki KL, Jenkins EL, Daya S, et al. Comparison of methods for image-based profiling of cellular morphological responses to small-molecule treatment. J. Biomol. Screen. 2013;18:1321–9.

Figure 1

Click here to download Figure Figure1.tif

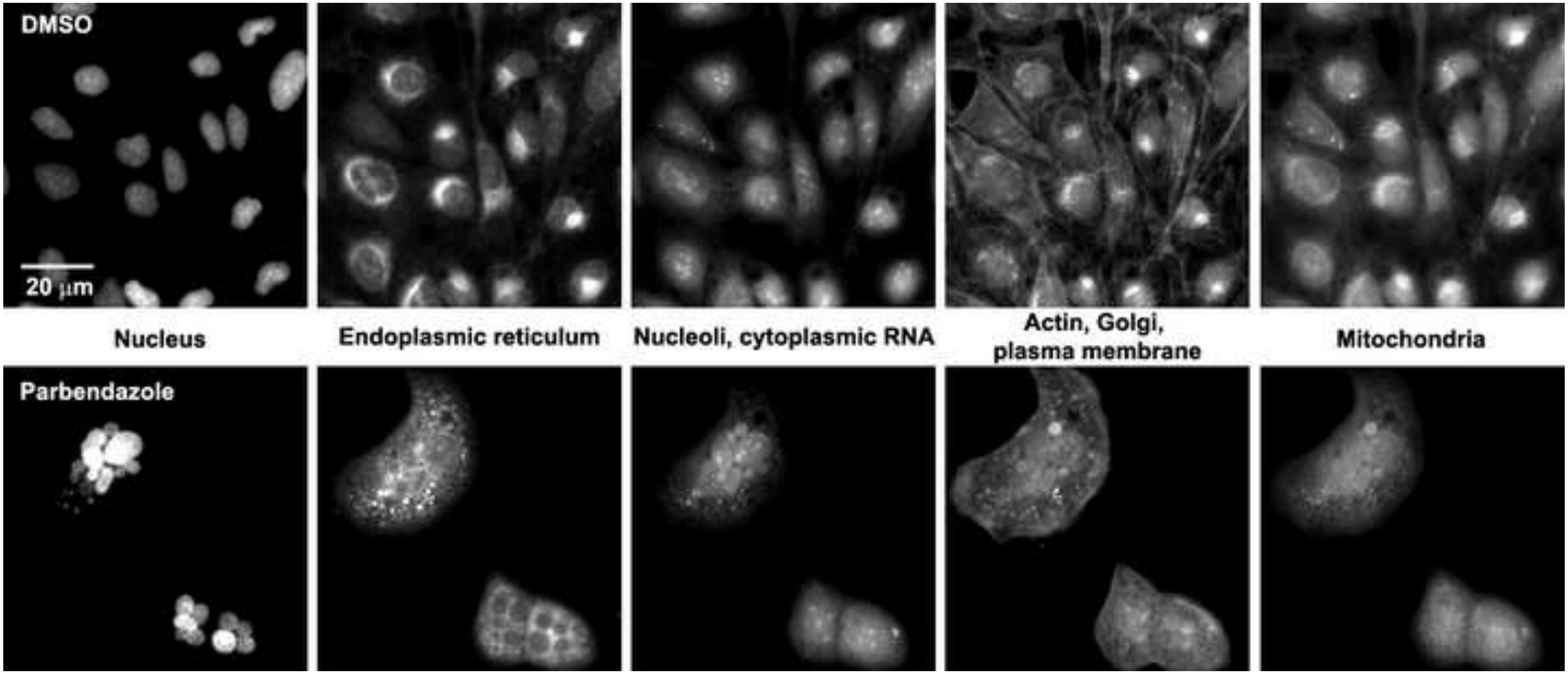

Supplement: GIGA-D-16-00012_Revision-2.pdf [file giw014_giga-d-16-00012_revision-2.pdf]
