# Supplementary material for: A dataset of images and morphological profiles of 30 000 small-molecule treatments using the Cell Painting assay
Source: Gigascience. 2017 Jan 7;6(12):1–5. doi: 10.1093/gigascience/giw014 (PMC5721342; doi:10.1093/gigascience/giw014)
Supplement: Reviewer-1_Original-Submission-(attachement).pdf [file giw014_reviewer-1_original-submission-(attachement).pdf]

Review report of the Data Note “A dataset of images and morphological profiles of 30,000 small-molecule treatments using the Cell Painting assay” by Bray, Gustafsdottir and Ljosa et al.

By Assaf Zaritsky, UTSW

In their Data Note “A dataset of images and morphological profiles of 30,000 small-molecule treatments using the Cell Painting assay” Bray, Gustafsdottir and Ljosa et al. report a publically available large scale data set of fixed single cell morphology features. The morphology of a cell and of its intracellular structures are established indicators for cell function in health and disease. A public dataset that may allow others to mine for their favorite compound, assess screening methods and study relations between different intra-cellular structures is definitely an important step in moving the field forward. Several organizations recognized this need and are currently investing considerable efforts in collecting and distributing such data; one example is the Alan institute for Cell Science to collect (live) intra-cellular measurements as a resource to study cell structure and the relations between its components. Another example is MULTIMOT and the Cell Migration Standardization Organization working to establish a common repository for cell imaging and migration data. As far as I am aware, the dataset reported here would be the largest of its kind that is publically available, only comparable in scale to Breinig and Klein et al. (MSB, 2015), and as such would be a valuable resource to the community.

Essential revisions:

1. The first paragraph in the section “Data acquisition protocol and quality control” reports the number of fluorescent dyes used (6) and the number of organelles imaged (8). It would be helpful to also state the number of imaged channels (5) at this stage. This is the one number of the three that is essential to understand the data; it is currently mentioned at the bottom of the next paragraph, which I find somewhat confusing.
2. The section “AVAILABILITY AND REQUIREMENTS” should refer to the current dataset and not to CellProfiler (listed in the next section).
3. There are several concerns in relation to data access and the ability to automate analysis.
    a. Discrepancy between plates numbers/names in GigaDB and The Cell Image library:
        i. Files in Giga DB: 24277-26796
        ii. The Image Cell Repository: 24278-26794
    b. I downloaded the data of plates 26796 and 26796 from GigaDB and examined a few entries at “The Cell Image Library” as test-cases:
        i. In http://www.cellimagelibrary.org/images/46295 the plate is annotated as 26680 (which do not even exist). Testing only a few plates, I will not be surprised this is not the only discrepancy and the authors should make sure that the data is correctly labeled.
        ii. In GigaDB, the file sizes of plates 26795 and 26796 are very different. I noticed in Plate_26796 – the ‘cells’ excel has no data in it. I also noticed

that there are other plates with the large variation in file size (e.g., 26785). Having 382 wells in each plate should make the total number of cells similar between plates and accordingly, the GigaDB file sizes. Or, do I miss something?

c. It will be very helpful for potential users of this data to be able to easily download, handle and accumulate it for their analyses. Some of the bullet points below are not absolutely essential, but can be very helpful.
    i. Can the authors provide means for mass download of the raw images? Currently, the plates can only be downloaded one-by-one from The Cell Image Library.
    ii. The 'cell' and 'image' tables are holding both the measurements (i.e., features) and some meta data (e.g., the Image_Metadata_Site column). It will be helpful to separate each to two tables.
    iii. Linking compound (treatment) to a well. I am aware that the Image_Metadata_BROAD_ID in the 'image' csv relates to the compound in the 'chemical_annotations' file but think that there could be a more intuitive way to make this link. One alternative would be to have another meta table that is dedicated to link these two.
    iv. I could not find which of the wells were controls for a given plate. This is a crucial piece of information that will allow the users to assess interday variation, and relate a phenotype to that day's controls. Admittedly, I have not tried very hard, so I might have missed it.

d. The scripts at https://github.com/CellProfiler/cytominr allow processing of accumulated well averages. Could be helpful to have a documentation of the available analyses at the supporting information. Also, if not present, could be helpful (not essential) to provide scripts that provide the population data, rather than the averages to enable easy post-processing.

Minor revisions and suggestions:

- Ideas for additional potential uses of this data set (Page 4, line #17):
    - Mine for morphological phenotypes of compounds as follow-ups to other compound-based screens
    - Allow others to assess screening methods and different morphological representations (e.g., see Gordonov etal. Integr. Biol., 2016)
    - Screen for compounds that alter the relations between the different intra-cellular structures imaged in this data set (e.g., alterations in spatial relations between cell nucleus and mitochondria)
- Single cell morphology profiling has many flavors and use examples in the literature. It would strengthen this Data Note to refer/cite some of this work to emphasize the potential broad usefulness of this resource.

- Page 2, line #7 (in Abstract): Typo, Perturbagen → perturbations
- Page 2, line #41: “Purpose of data acquisition”, can you find a better heading?
- Page 3 top: “using statistical analysis to uncover latent morphological patterns”, ‘latent’ is probably not the best word here.
- Page 3, line #29: would be better to have the references [8,9] cited at the first paragraph.
- Page 3, line #38: “diversity-oriented synthesis (DOS)” – this term is mentioned only once in the manuscript and so should not be abbreviated.
- Page 4, line #1: I would refer again to Additional File 1 (for the description of the features extracted).
- Page 4, line #5: “The pipelines, ICFs, and extracted morphological data are provided the GigaDB repository.”. Missing the word “in”?
- Page 4, line #47: Provide ‘The Cell Images Library’ URL and its specific accession numbers (CIL: 24278- CIL: 26794) in addition to the plates #.
- Page 4, line #57: Naming of the Cell Painting wiki includes the “natprot” suffix which the authors might want to update https://github.com/carpenterlab/2016_bray_natprot
- Page 8, line #9 (Table 2):
  - Would be helpful to mention the number of wells per plate in the Description.
  - Reporting the physical pixel size could be very useful for researchers that would like to re-analyze this data.
- When extracting the compressed plate information from the GigaDB, the files are extracted to the directory broad\hptmp\mbray\gigascience\to_archive\Plate_XXXXX. Is it really needed to have this entire path?

Disclaimer: In reviewing the manuscript I have not executed the scripts provided by the authors. I trust that they perform as reported.
