# Supplementary material for: A dataset of images and morphological profiles of 30 000 small-molecule treatments using the Cell Painting assay
Source: Gigascience. 2017 Jan 7;6(12):1–5. doi: 10.1093/gigascience/giw014 (PMC5721342; doi:10.1093/gigascience/giw014)
Supplement: Response-to-Reviewer-Comments_Original-Submission.pdf [file giw014_response-to-reviewer-comments_original-submission.pdf]

We thank the reviewers for their helpful comments; it is clear they made a careful review of the data in detail and we are very impressed and grateful. The editor made a number of comments concerning formatting which we have addressed in the revised text. Below, we respond to each of the points that the reviewers raised.

Reviewer #2

I had some problems downloading the files from my Mac using both the Finder and using a Python script. I suspect that the files were uploaded using ASCII instead of binary, which can corrupt the files during download. I did manage in the end to access the files using a Windows machine and FileZilla. Maybe the authors could test the download from different platforms to see whether they can reproduce my issues and if yes, provide instructions as to what solution works.

Thank you for pointing out this issue. We will work with the Gigascience curators to ensure that the contributed data has its integrity preserved during transfer.

Reviewer #3

The image data presented in the Cell Image Library (reference 10) does not include all of the plate images described in the GigaDB submission. My efforts to resolve this, using the plate list in the image_curation_statistics.csv file found 60 plate IDs that did not have a corresponding image set (the list of plate IDs that lacked image data is attached *).

(A similar point was also noted by Reviewer #2, so we address both here)

We are grateful the reviewers noticed this problem. The original Cell Painting dataset contained plates that were imaged but not uploaded to CIL. We have resolved this discrepancy by contributing the additional image data to CIL.

The pipelines in the GigaDB submission were designed for an earlier version of CellProfiler. The versions on the Cell Painting Wiki were up to date and should be included in the final submission to GigaDB.
The pipelines did not work correctly with the image data. In particular, the regular expression parser for metadata in the file directory path was designed for a different file format. This should be updated to work with the image sets deposited on cellimagelibrary.org (reference 10).

Indeed, the pipelines on the Cell Painting wiki are adapted to image sets from a different paper (i.e, Singh et al 2015) which have slightly different image metadata than the images in The Cell image library. We will be keeping those pipelines continually updated going forward, and thus it is still quite valuable to link to them even though there is a mismatch in metadata. We think that providing multiple versions of the pipelines, each suited to a different image set could cause confusion and more work in keeping them all updated properly. Therefore, we decided to add text to the Additional File to describe how the pipelines at that location must be adapted to work for the images from The Cell image library.
Note that in the resubmission, we also have ensured that the pipelines provided as supplementary data will work directly with images at The Cell library. These pipelines use CellProfiler's regular Input modules and are suited for a small number of images (which can be dragged and dropped into CellProfiler's interface) We also provide instructions on how to adapt these pipelines to use the LoadData module which is suited for a large batch of images where loading all filenames would otherwise take an inordinate amount of time.
Reviewer #4

Essential revisions:
The first paragraph in the section "Data acquisition protocol and quality control" reports the number of fluorescent dyes used (6) and the number of organelles imaged (8). It would be helpful to also state the number of imaged channels (5) at this stage. This is the one number of the three that is essential to understand the data; it is currently mentioned at the bottom of the next paragraph, which I find somewhat confusing.

Good idea. We have added the phase "..., and imaged with five fluorescent channels (Fig. 1)" to the end of the paragraph mentioned.

The section "AVAILABILITY AND REQUIREMENTS" should refer to the current dataset and not to CellProfiler (listed in the next section).

Since we have not included code as part of this submission, we have removed this section.

I downloaded the data of plates 26796 and 26796 from GigaDB and examined a few entries at "The Cell Image Library" as test-cases:
In http://www.cellimagelibrary.org/images/46295 the plate is annotated as 26680 (which do not even exist). Testing only a few plates, I will not be surprised this is not the only discrepancy and the authors should make sure that the data is correctly labeled.

Thank you for pointing us to this discrepancies. For the final version, we have cross-checked our references to what is posted.

In GigaDB, the file sizes of plates 26795 and 26796 are very different. I noticed in Plate_26796 – the 'cells' excel has no data in it.

The reviewer is correct in pointing out that some plates had no per-cell data. Since this submission, we have tracked down the omitted cellular data and included it in the final GigaDB posting.

I also noticed that there are other plates with the large variation in file size (e.g., 26785). Having 382 wells in each plate should make the total number of cells similar between plates and accordingly, the GigaDB file sizes. Or, do I miss something?

Regrettably, not all images could be recovered from our data archives, and hence not all plates are complete. However, we felt that even partial plates would still be a valuable contribution for public dissemination, provided it was annotated accordingly. This information regarding archive completeness is recorded in image_curation_statistics.csv, mentioned in Table 2 and is also posted at GigaDB. We have added a mention of this file in the "Availability of supporting data" section.

It will be very helpful for potential users of this data to be able to easily download, handle and accumulate it for their analyses. Some of the bullet points below are not absolutely essential, but can be very helpful.
Can the authors provide means for mass download of the raw images? Currently, the plates can only be downloaded one-by-one from The Cell Image Library.

We have provided a bash shell script to facilitate downloading the entire image set from CIL in batch.

The 'cell' and 'image' tables are holding both the measurements (i.e., features) and some meta data (e.g., the Image_Metadata_Site column). It will be helpful to separate each to two tables.
Linking compound (treatment) to a well. I am aware that the Image_Metadata_BROAD_ID in the 'image' csv relates to the compound in the 'chemical_annotations' file but think that there could be a more intuitive way to make this link. One alternative would be to have another meta table that is dedicated to link these two.
I could not find which of the wells were controls for a given plate. This is a crucial piece of information that will allow the users to assess interday variation, and relate a phenotype to that day's controls. Admittedly, I have not tried very hard, so I might have missed it.

We appreciate the above three comments pointing out how the metadata could be better organized. The details on which columns contain the compound information were contained in the image tables, and were listed in the Additional File, under "Extracted morphological features"; however, we agree that this organization is confusing. We have added an additional table for each plate containing the metadata, with references to these tables mentioned in the main text and the Additional File under "Extracted morphological features" and "Chemical compound annotations".

The scripts at https://github.com/CellProfiler/cytominr allow processing of accumulated well averages. Could be helpful to have a documentation of the available analyses at the supporting information.

We have included mention of the cytominR package in the "Data acquisition protocol and quality control" and "Available of supporting data" sections.

Also, if not present, could be helpful (not essential) to provide scripts that provide the population data, rather than the averages to enable easy post-processing.

The per-cell data (what we think the reviewer means here by "population data") is in fact the raw data that

is provided as this data set ("Extracted morphological features" in Table 2); no scripts are needed to provide them.

Minor revisions and suggestions:

Ideas for additional potential uses of this data set (Page 4, line #17):
Mine for morphological phenotypes of compounds as follow-ups to other compound-based screens
Allow others to assess screening methods and different morphological representations (e.g., see Gordonov etal. Integr. Biol., 2016)
Screen for compounds that alter the relations between the different intra-cellular structures imaged in this data set (e.g., alterations in spatial relations between cell nucleus and mitochondria)

We appreciate these additional ideas and have included them in the text.

Single cell morphology profiling has many flavors and use examples in the literature. It would strengthen this Data Note to refer/cite some of this work to emphasize the potential broad usefulness of this resource.

We agree that given the wide-ranging nature of the field, additional citations are warranted. For the sake of brevity, we point the reader to a recent (2016) review of image-based profiling considerations by Caicedo et al.

Page 2, line #7 (in Abstract): Typo, Perturbagen → perturbations

We have changed the wording here.

Page 2, line #41: "Purpose of data acquisition", can you find a better heading?

We have replaced this heading with "Background".

Page 3 top: "using statistical analysis to uncover latent morphological patterns", 'latent' is probably not the best word here.

We have replaced "latent" with "subtle".

Page 3, line #29: would be better to have the references [8,9] cited at the first paragraph.
These two references are now mentioned in the 1st paragraph.

Page 3, line #38: "diversity-oriented synthesis (DOS)" – this term is mentioned only once in the manuscript and so should not be abbreviated.

We have removed the abbreviation.

Page 4, line #1: I would refer again to Additional File 1 (for the description of the features extracted).

A reference to Additional File 1 has been added.

Page 4, line #5: "The pipelines, ICFs, and extracted morphological data are provided the GigaDB repository.". Missing the word "in"?

The typo has been fixed.

Page 4, line #47: Provide 'The Cell Images Library' URL and its specific accession numbers (CIL: 24278-CIL: 26794) in addition to the plates #.

Accession numbers have been added.

Page 8, line #9 (Table 2):
Would be helpful to mention the number of wells per plate in the Description.
Reporting the physical pixel size could be very useful for researchers that would like to re-analyze this data.

The number of wells and the pixel size has been added.

When extracting the compressed plate information from the GigaDB, the files are extracted to the directory broad\hptmp\mbray\gigascience\to_archive\Plate_XXXXX. Is it really needed to have this entire path?

The reviewer is correct: It is not necessary to have this path; we apologize for this inconvenience. The compressed files in each archive are now extracted to the location specified by the user, under a folder named with the plate identifier.

Page 4, line #57: Naming of the Cell Painting wiki includes the "natprot" suffix which the authors might want to update https://github.com/carpenterlab/2016_bray_natprot

A manuscript describing the cell painting protocol in full is in press at Nature Protocols; the wiki nomenclature refers to links provided in that draft.
