# Supplementary material for: A dataset of images and morphological profiles of 30 000 small-molecule treatments using the Cell Painting assay
Source: Gigascience. 2017 Jan 7;6(12):1–5. doi: 10.1093/gigascience/giw014 (PMC5721342; doi:10.1093/gigascience/giw014)
Supplement: Response-to-Reviewer-Comments_Revisions-1.pdf [file giw014_response-to-reviewer-comments_revisions-1.pdf]

Revised version with data sections formatted correctly here.
