# Supplementary material for: A dataset of images and morphological profiles of 30 000 small-molecule treatments using the Cell Painting assay
Source: Gigascience. 2017 Jan 7;6(12):1–5. doi: 10.1093/gigascience/giw014 (PMC5721342; doi:10.1093/gigascience/giw014)
Supplement: Reviewer-2-Report-(Original-Submission).pdf [file giw014_reviewer-2-report-(original-submission).pdf]

Reviewer Report

Title: "A dataset of images and morphological profiles of 30,000 small-molecule treatments using the Cell Painting assay"

Version: Original Submission **Date:** 3/14/2016

Reviewer name: Marc Bickle

Reviewer Comments to Author:

The manuscript of Bray et al submits the data to their previously published Cell Painting assay. The execution of the assay and the analysis of the assay are described in sufficient details elsewhere that scientists accessing the data should be able to reproduce the data. This is a very important resource for the community, as it represents a benchmark for developing novel analytical tools to profile experimental conditions. The approach of profiling phenotypes to understand function is as old as Mendel's finding and are unquestionably valuable. The Cell Painting assay is continuation of the technique of observation using modern computer and mathematical tools. The analysis of this type of dataset is a very active field and having an extensive dataset for benchmarking is extremely valuable.

I had some problems downloading the files from my Mac using both the Finder and using a Python script. I suspect that the files were uploaded using ASCII instead of binary, which can corrupt the files during download. I did manage in the end to access the files using a Windows machine and FileZilla. Maybe the authors could test the download from different platforms to see whether they can reproduce my issues and if yes, provide instructions as to what solution works. This is my only comment for changes to the text.

Level of Interest

Please indicate how interesting you found the manuscript: An article of importance in its field

Quality of Written English

Please indicate the quality of language in the manuscript: Needs some language corrections before being published

Declaration of Competing Interests

Please complete a declaration of competing interests, considering the following questions:

- Have you in the past five years received reimbursements, fees, funding, or salary from an organisation that may in any way gain or lose financially from the publication of this manuscript, either now or in the future?

- Do you hold any stocks or shares in an organisation that may in any way gain or lose financially from the publication of this manuscript, either now or in the future?
- Do you hold or are you currently applying for any patents relating to the content of the manuscript?
- Have you received reimbursements, fees, funding, or salary from an organization that holds or has applied for patents relating to the content of the manuscript?
- Do you have any other financial competing interests?
- Do you have any non-financial competing interests in relation to this paper?

If you can answer no to all of the above, write 'I declare that I have no competing interests' below. If your reply is yes to any, please give details below.

I declare that I have no competing interests

I agree to the open peer review policy of the journal. I understand that my name will be included on my report to the authors and, if the manuscript is accepted for publication, my named report including any attachments I upload will be posted on the website along with the authors' responses. I agree for my report to be made available under an Open Access Creative Commons CC-BY license (http://creativecommons.org/licenses/by/4.0/). I understand that any comments which I do not wish to be included in my named report can be included as confidential comments to the editors, which will not be published.

I agree to the open peer review policy of the journal
